# Medical English anxiety patterns among medical students in Sichuan, China

**DOI:** 10.3389/fpsyg.2022.895117

**Published:** 2022-08-10

**Authors:** Jiaqi Deng, Kaiji Zhou, Ghayth K. S. Al-Shaibani

**Affiliations:** ^1^School of Foreign Languages, Southwest Medical University, Luzhou, China; ^2^Department of Education, Faculty of Social Sciences and Liberty Arts, UCSI University, Kuala Lumpur, Malaysia; ^3^Department of Applied Social Sciences, The Hong Kong Polytechnic University, Hong Kong, China

**Keywords:** anxiety scale for learning, English for specific purposes (ESP), medical English, Chinese medical students, Medical English Language Anxiety Scale, medical English language anxiety

## Abstract

This study adapts a Medical English Language Anxiety Scale (MELAS) based on Horwitz’s Foreign Language Classroom Anxiety Scale (FLCAS) and examines students’ anxiety in medical English vocabulary, listening and speaking, communication, literature reading, and academic paper writing. The biographical factors related to medical English language anxiety (MELA) were also tested. The questionnaire sets including five dimensions were distributed to the students from a medical university in Sichuan, China, and were statistically analyzed by using SPSS 22.0 and AMOS 21.0. By employing the adapted MELAS, it was found that 85.2% of the medical students surveyed suffered moderate and higher anxiety. Among all dimensions, students with listening and speaking anxiety recorded the highest (89.3%), followed by literature reading anxiety (86.6%), English academic writing anxiety (85.9%), communication anxiety (81.9%), and vocabulary learning anxiety (81.2%). We also found that the anxiety of rural medical students in each dimension was higher than that of urban medical students. This study suggests that English teachers should be fully aware of their students’ language anxiety situation, design interesting class activities, and create a relaxed English learning atmosphere in classroom teaching to make students less nervous when learning medical English in class.

## Introduction

Anxiety has been described as the subjective feeling of tension, apprehension, nervousness, and worry associated with arousal of the autonomic nervous system (Spielberger, 1983). Since the 1970s, anxiety has been one of the most important affective factors affecting language development (Arnold, 2005), gradually becoming an important part of second language acquisition research in China and abroad (Guo and Xu, 2014). Second language researchers and theorists have long been aware that anxiety is often associated with language learning. Horwitz et al. (1986, pp. 125–132) first put forward “foreign language anxiety (FLA),” defined it as “a distinct complex of self-perceptions, beliefs, feelings, and behaviors related to classroom language learning arising from the uniqueness of the language learning process,” and then developed the foreign language classroom anxiety scale (FLCAS), which is the most widely used foreign language anxiety testing tool (Luo, 2013). Then, a large number of studies (Xiong, 2007; Awan et al., 2010; Liu, 2011; Ghorban Dordinejad and Nasab, 2013; Al-Shboul et al., 2013; Shao et al., 2013; Cakici, 2016; Sajid, 2018; Du, 2019) adopted the FLCAS to test the correlation between foreign language anxiety and language achievement.

In college English education in today’s China, English for General Purposes (EGP) and English for Special Purposes (ESP) coexist in the college English teaching system (Wen, 2014). The way out and developing direction of college English teaching in China is ESP, and college English teaching policy should be transferred from EGP to ESP (Cai, 2014). Thus, ESP is receiving more attention, especially medical English. Chinese medical students, as a highly professional group, need to publish their research findings in Science Citation Index (SCI) journals abroad according to the current requirements of master’s degree and doctor’s training and doctor’s promotion system; that is, they must be able to read the literature and write research papers in English. Therefore, English is more important to these students than any other major. Only a few studies (Dong, 2002; Wang et al., 2009; Tong and Zang, 2012; Li, 2018) have conducted relevant research on this topic, and they adopted the FLCAS to test medical English anxiety. Hence, it is necessary to explore medical students’ medical English anxiety. The present study thus aims to identify the following three research questions:

1. What is the overall situation of medical students’ MELA by using the adapted MELAS?
2. How do the biographical factors affect medical students’ MELA?
3. How does students’ MELA correlate with their language achievement?

## Literature review

### English education in Chinese universities

English is a public language in the world, and learning English is a “world language fashion” gradually formed since World War II (Li and Tang, 2020, pp. 17–30). In 2008, Euronews reported “at present, there are approximately 250 million people learning English in China, which is the largest number of people in a country learning English in the world” (Zhang, 2015, pp. 56–60). English is a compulsory course for all Chinese university students. After the promulgation of the National Standard for Teaching Quality of Foreign Language Majors in Colleges and Universities and the formulation of the College English Curriculum Requirements, a series of foreign language teaching reforms were carried out in colleges and universities across the country. One of the main directions is to shift from traditional English for general purposes (EGP) to English for specific purposes (ESP) and English for academic purposes (EAP) (Tao and Gao, 2019). General English (GE) is mainly based on language proficiency (Yao and Wang, 2020), while ESP includes not only specific language skills but also professional content, aiming at cultivating students’ English application ability in specific professional fields (Liu and Du, 2018). Ma and Xia (2014) proposed that teaching should also include professional knowledge rather than only focusing on language knowledge.

With China’s opening up to the outside world and the growth of the economy and society, the development of medical and health services also presents unprecedented prosperity. The exchange and communication of medical affairs around the world are increasingly happening. English is widely used in the medical field. Many medical students and medical staff realize the importance of medical English learning (Huang, 2014). Ma (2009) pointed out that medical English should be the most important component of college English teaching in medical colleges and universities; Ma added that the combination of EGP and medical knowledge can yield fruitful outcomes for medical students in China.

### Foreign language anxiety and foreign language classroom anxiety scale: Anxiety scales

Since the 1980s, the study of second language acquisition has made great progress. Some studies have focused on the relation between affective variables and foreign language learning (Gardner, 1985; Steinberg and Horwitz, 1986; Young, 1992). Anxiety is considered to be one of the most critical psychological variables, and language anxiety is a complex psychological phenomenon peculiar to language learning (Wang and Wan, 2001). However, in the 1980s, Horwitz et al. (1986) found that few studies have looked at subtle effects of anxiety on foreign language learning due to the limitations of measuring instruments. The first anxiety scale to measure foreign language learning was the French Classroom Anxiety Scale, designed by Gardner et al. (1976) to measure French foreign language anxiety (FLA). Ely (1986) constructed the Language Classroom Discomfort Scale to investigate students’ anxiety, self-awareness, or embarrassment when speaking a foreign language in class. Based on previous research, Horwitz et al. (1986) developed the Foreign Language Classroom Anxiety Scale (FLCAS). This scale has demonstrated internal reliability, achieving an alpha coefficient of (0.93) with all items producing significant corrected item-total scale correlations. This scale is widely used to measure foreign language anxiety.

Since the foreign language classroom anxiety scale has become a tool to measure anxiety, most studies have pointed out that there is a significant negative correlation between foreign language anxiety and foreign language achievement (Horwitz, 2001; Hu, 2016; Li, 2020; Dong, 2021). Many different scales were adapted from the FLCAS by some researchers; however, these adapted scales only measured one dimension of students’ English competency, such as listening scale, writing scale, and speaking scale (Saito et al., 1999; Kim, 2000; Wu, 2008; Bashir, 2014).

Research on Chinese students’ medical English anxiety is rare. Zhao et al. (2018) studied the *status quo* of medical English listening anxiety of nursing undergraduates and found that the 84.2% of the surveyed students had medical English listening anxiety at different degrees; Wu et al. (2012) conducted a survey of oral English anxiety of medical college students. They found that 71.5% medical students suffer moderate and high anxiety in their oral medical English learning, among which male students were more anxious than female students; more suburban students suffered moderate and high anxiety than urban students. Furthermore, these studies were conducted on medical language anxiety, but anxiety was tested by using FLCAS (Wu et al., 2012; Li, 2014; Zhao et al., 2018). We hold that it is not persuasive to test the language anxiety of students in a specific profession because learning ESPs is more difficult than English for General Purposes (EGPs). In addition to memorizing basic EGPs rules, grammar and complicated professional terms, students also need to memorize and understand sentence structures and writing patterns in ESPs. To support this argument, Shi (2014) made a comparison of students’ anxiety in learning EGP and ESP and found that there was a significant difference in students’ anxiety level when learning ESP and EGP. This is due to some factors such as different teaching contents and requirements for students in ESP and EGP, students’ learning motivation, and study burden differences which contributed to affect students’ ESP and EGP learning anxiety. We argue that it is better to have specific scales to measure the specific anxiety according to different majors. Therefore, we adapted a specific Medical English Language Anxiety Scale (MELAS) from FLCAS to measure Chinese medicos’ medical English language anxiety.

### Sources of foreign language anxiety

Foreign language anxiety was found to be associated with a variety of independent variables. Jiang and Dewaele (2020) explored the link between Chinese students’ FLA in English and associated socio-biographical variables (i.e., gender, ethnic group affiliation, geographical background, and experience traveling abroad) and socio-biographical and language variables (i.e., age of onset of acquisition, language achievement level, self-perceived oral competence, and frequency of language use). Russell (2020) examined language anxiety in the context of an online learning environment. Young (1991) defined six potential sources of language anxiety: (a) personal and interpersonal anxieties, (b) learner beliefs about language learning, (c) instructor beliefs about language teaching, (d) instructor-learner interactions, (e) classroom procedures, and (f) language testing.

The effect of gender on FLA has not reached a consensus. Some studies found no gender differences (Matsuda and Gobel, 2004; Garcia de Blakeley et al., 2017); while other studies found that female learners experienced more FLA than their male counterparts (Ezzi, 2012; Park and French, 2013; Gargalianou et al., 2015). However, Dewaele et al. (2019) found that male Kazakh learners of Turkish experienced higher levels of FLA in the classroom than their female peers. In Elaldi’s (2016) study, foreign language anxiety among males was found to be higher than that among females for Turkish students learning English.

Regarding geographical background, Jiang and Dewaele (2020) found that geographical background has a significant effect on participants’ FLA in two situations (when speaking English with friends as well as with classmates). Wang and Ding (2001) found that the conditions of learning English for students in western remote rural areas of China are far inferior to those in developed urban areas, and thus rural students have more serious foreign language anxiety.

Based on the literature review, the researchers of this study did not find a study on students’ medical English anxiety by using a specific medical English anxiety scale. In addition, the relationship of factors with language anxiety was not clear.

## Materials and methods

### Participants

The survey was conducted on medical university students in Sichuan Province, China. A total of 212 questionnaire sets were distributed and 191 questionnaire sets were returned. Among the returned 191 sets, the incomplete sets were disregarded, and 149 sets completed in full were used in the study and they are valid. This includes 69 male students and 80 female students (57 urban students and 92 suburban students). In terms of their score, 86 top students (with a score range of 80–90), 60 average students (with a score range of 60–80), and 3 failed students (with a score of 60). Therefore, all the subjects included in this study were mainly composed of average junior students and above, with an even distribution of gender.

### Research instrument

The language anxiety scale for medical students used in this study was adapted from Guo and Wu’s (2008) revised version of Horwitz’s FLCAS. This adapted medical English language anxiety scale (MELAS) includes five dimensions: ① medical English vocabulary learning anxiety, ② medical English listening and speaking anxiety, ③ medical English communication anxiety, ④ medical English literature reading anxiety, and ⑤ medical English academic paper writing anxiety which work together to reveal medical English anxiety. We designed a questionnaire consisting of 25 items and used a 5-point Likert scale. The score of the questionnaire is the cumulative total score, and the theoretical score range is 18–90 points. If the participants received higher scores, they suffered more serious anxiety in medical English language learning. The questionnaire language is Chinese, given that the participants have differing proficiencies in English which may affect the results.

### Data collection

The convenience sampling method was adopted in this study. All four classes taught by the first author in a medical university in Sichuan, China, were included and all 212 students from those 4 classes were chosen as the participants. A total of 212 sets of questionnaire were distributed to them and collected in the classroom by the first author. The questionnaire sets were completed in the traditional paper-and-pencil way with their consent. The participants spent 15–20 min to complete their response and they took part in this study anonymously. Of the 212 sets of questionnaire distributed to the participants, 191 sets were returned. For the return rate, it was 90%. Among the returned 191 sets, the incomplete sets were disregarded, and only 149 sets completed in full were used in this study. The data of 149 participants were thus analyzed in this study.

### Data analysis

The students spent approximately 15–20 min filling in the MELAS questionnaire after their English final examination in June 2020. Then, the data were statistically analyzed by using SPSS 22.0 and AMOS 21.0.

## Results

We tested the adapted questionnaire’s reliability and validity by measuring its Cronbach’s α alpha by using second-order confirmatory factor structural equation model first. After proving the adapted scale is reliable and valid, we then applied this scale to measure the participants’ general condition of medical English anxiety so as to address the first research question. As for the independent sample *t*-test, one-way by variance analysis and multiple logistic regression analysis were performed on the relation of demographic variables with learning anxiety, and English scores with anxiety so as to address the second and the third research questions.

### Reliability analysis

Cronbach’s α alpha of the questionnaire was 0.918 and that of each subscale was between 0.700 and 0.843, indicating that the internal consistency reliability of this scale was very high in this study as shown in Table 1.

**TABLE 1:** Scale reliability analysis.

Dimension	Cronbach’s alpha	Number of items
1. Vocabulary learning anxiety	0.843	3
2. Listening and speaking anxiety	0.812	5
3. Communication anxiety	0.700	3
4. Literature reading anxiety	0.772	4
5. English academic paper writing anxiety	0.783	3
Scale:	0.918	18

### Validity analysis

This scale was divided into five anxiety dimensions, namely, ① vocabulary learning anxiety, ② listening and speaking anxiety, ③ communication anxiety, ④ literature reading anxiety, and ⑤ English academic paper writing anxiety, reflecting an overall medical English language anxiety situation among medical students. Therefore, second-order confirmatory factor analysis was used to analyze the validity of the questionnaire.

**FIGURE 1:**
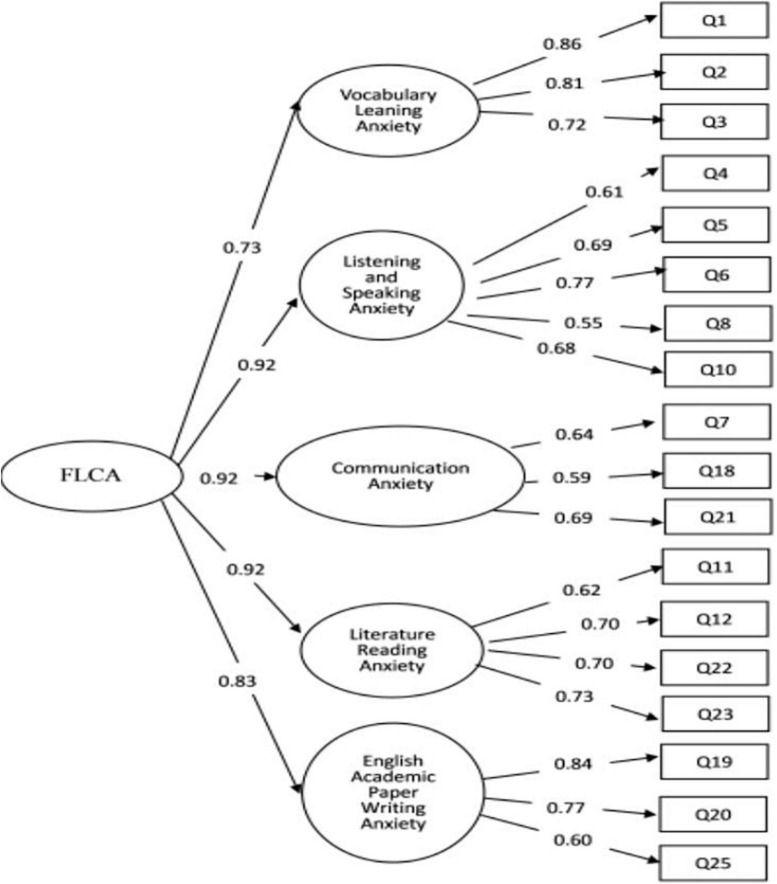
Second-order confirmatory factor analysis (structural equation model).

A second-order confirmatory factor structural equation model was constructed on the collected scale according to the pre-divided dimensions. Then, based on the calculation results of the initial model, the result of the CFA of scale is obtained after modifying the model without violating the experience and theory, as shown in the figure below (Figure 1).

The matching of the above verification factor analysis model and the data of the scale is good (χ^2^/*df* < 3, RMSEA < 0.08, IFI > 0.9, TLI > 0.9, CFI > 0.9). The matching index values are shown in Table 2.

**TABLE 2:** Scale validation factor analysis model compatibility.

	χ^2^/*df*	RMSEA	GFI	IFI	TLI	CFI
Model	1.723	0.070	0.870	0.938	0.913	0.936

It can be clearly seen based on the fit index of Table 2 that the second-order confirmatory factor model of the scale matches the scale’s data very well. χ^2^/*df* < 3, RMSEA < 0.08, IFI > 0.9, TLI > 0.9, CFI > 0.9, indicating that the scale has good validity (Byrne, 2013).

From the above analysis of the convergent validity of the scale, it can be seen that the factor load of the 18 items in the first-order dimension is between 0.5 and 0.99 which is the same as the factor load of the five anxiety dimensions in the second-order dimension of the general emotion. Therefore, among the original 25 items, these 18 items whose factor load is between 0.5 and 0.99 are finally set. In addition, as shown in Table 3, the combination reliability of the five anxiety dimensions and the general sentiment of the second-order dimension are all above 0.6, indicating that the internal quality of the scale is very good (Bentler, 2009). Furthermore, the average variance extraction (AVE) of the five anxiety dimensions of the scale and the second-order dimensional anxiety are all above 0.4, indicating that the aggregation and convergence validity of the scale is good (Fornell and Larcker, 1981).

**TABLE 3:** Scale convergence validity analysis.

Dimension	Item	Factor load	Reliability coefficient	Measurement error	Combination reliability CR	Average variance extraction AVE
Anxiety	Vocabulary learning anxiety	0.729	0.531	0.469	0.937	0.750
	Listening and speaking anxiety	0.919	0.845	0.155		
	Communication anxiety	0.924	0.854	0.146		
	Literature reading anxiety	0.916	0.839	0.161		
	English academic paper writing anxiety	0.826	0.682	0.318		
Vocabulary learning anxiety	Q1	0.858	0.736	0.264	0.841	0.640
	Q2	0.806	0.650	0.350		
	Q3	0.730	0.533	0.467		
Listening and speaking anxiety	Q4	0.605	0.366	0.634	0.793	0.436
	Q5	0.685	0.469	0.531		
	Q6	0.765	0.585	0.415		
	Q10	0.676	0.457	0.543		
	Q8	0.552	0.305	0.695		
Communication anxiety	Q7	0.636	0.404	0.596	0.671	0.519
	Q18	0.585	0.342	0.658		
	Q21	0.693	0.480	0.520		
Literature reading anxiety	Q11	0.621	0.386	0.614	0.783	0.476
	Q12	0.703	0.494	0.506		
	Q22	0.700	0.490	0.510		
	Q23	0.73	0.533	0.467		
Anxiety in English academic writing	Q19	0.844	0.712	0.288	0.786	0.556
	Q20	0.774	0.599	0.401		
	Q25	0.597	0.356	0.644		

Based on all the analysis results above, the scale is reliable and valid. It is appropriate to divide the 18 items in the scale into five anxiety dimensions which reveal the anxiety state very accurately (Hair et al., 2013).

### General condition of medical English learning anxiety for medical students

To understand the distribution of students’ anxiety, students were divided into a high anxiety group, middle anxiety group, and low anxiety group according to the total anxiety score and the average score of five dimensions. Those who scored one standard deviation higher than the average score were classified as the high anxiety group, those who scored one standard deviation lower than the average score were classified as the low anxiety group, and the rest were classified as the middle anxiety group (Table 4).

**TABLE 4:** Scale score.

Scale	*M* ± *SD*
1. Vocabulary learning anxiety	3.47 ± 1.06
2. Listening and speaking anxiety	3.44 ± 0.88
3. Communicative anxiety	3.27 ± 0.88
4. Literature reading anxiety	3.480 ± 0.83
5. English academic writing anxiety	3.58 ± 0.92
Overall	3.45 ± 0.74

As shown in Table 5, 85.2% of the students surveyed had a moderate score and above. Among all dimensions, students with listening and speaking anxiety accounted for the highest proportion (89.3%), followed by literature reading anxiety (86.6%), English academic writing anxiety (85.9%), communication anxiety (81.9), and vocabulary learning anxiety (81.2%).

**TABLE 5:** Distribution of samples at each latitude.

	Total anxiety score		Vocabulary learning anxiety		Listening and speaking anxiety		Communication anxiety		Literature reading anxiety		English academic papers writing anxiety	
						
	*n*	Percentage	*n*	Percentage	*n*	Percentage	*n*	Percentage	*n*	Percentage	*n*	Percentage
High anxiety	23	15.4%	31	20.8%	24	16.1%	21	14.1%	19	12.8%	26	17.4%
Middle anxiety	104	69.8%	90	60.4%	109	73.2%	101	67.8%	110	73.8%	102	68.5%
Low anxiety	22	14.8%	28	18.8%	16	10.7%	27	18.1%	20	13.4%	21	14.1%

### Analysis of variance of demographic variables and learning anxiety

Taking gender and geographical background as independent variables and each dimension as the dependent variable, an independent sample *t*-test was performed. The results are shown in Tables 6, 7.

**TABLE 6:** Gender difference test of medical students’ English learning anxiety.

	Male (*n* = 69)	Female (*n* = 80)	*t*	*p*
Vocabulary learning anxiety	10.74 ± 3.48	10.15 ± 2.91	1.126	0.262
Listening and speaking anxiety	17.06 ± 4.68	17.39 ± 4.19	−0.454	0.651
Communication anxiety	9.43 ± 2.59	10.15 ± 2.68	−1.650	0.101
Literature reading anxiety	13.88 ± 3.25	13.95 ± 3.45	−0.120	0.905
Essay writing anxiety	10.51 ± 2.94	10.96 ± 2.60	−1.004	0.317
Overall anxiety	61.62 ± 14.09	62.60 ± 12.67	−0.446	0.657

**TABLE 7:** Geographical backgrounds test in English learning anxiety of medical students.

	Urban (*n* = 57)	Rural (*n* = 92)	*t*	*p*
Vocabulary learning anxiety	9.54 ± 3.10	10.97 ± 3.14	−2.705	0.008
Listening and speaking anxiety	16.09 ± 4.51	17.95 ± 4.22	−2.546	0.012
Communication anxiety	9.07 ± 2.39	10.28 ± 2.71	−2.771	0.006
Literature reading anxiety	12.79 ± 3.45	14.62 ± 3.09	−3.357	0.001
Essay writing anxiety	9.98 ± 2.84	11.23 ± 2.61	−2.735	0.007
Overall anxiety	57.47 ± 13.65	65.04 ± 12.29	−3.501	0.001

From the data in Table 6, the scores of male students in the vocabulary learning anxiety dimension were slightly higher than those of female students, but the difference was not statistically significant. The scores of girls in other dimensions were higher than those of boys, but the differences were not statistically significant either.

According to the data in Table 7, the anxiety scores of rural medical students in each dimension were higher than those of urban medical students, and the differences in each dimension were significant.

**FIGURE 2:**
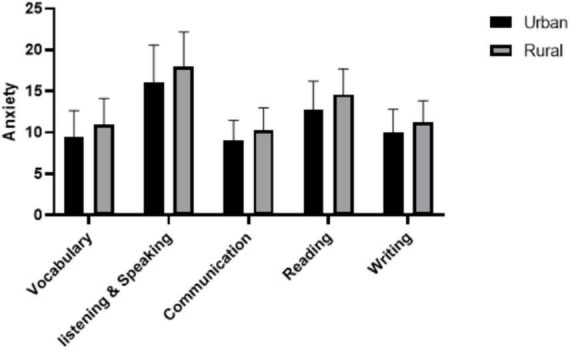
Comparison of urban and rural students’ foreign language anxiety.

One-way analysis of variance was performed with grades and scores as independent variables and each dimension as a dependent variable. The results are shown in Tables 8, 9 (Figure 2).

**TABLE 8:** Score difference in language achievement.

	Excellent (*n* = 86)	Medium (*n* = 60)	Failed (*n* = 3)	*F*	*p*
Vocabulary learning anxiety	9.77 ± 3.12	11.17 ± 3.07	14.33 ± 1.15	6.100	0.003
Listening and speaking anxiety	16.70 ± 4.14	17,67 ± 4.60	24.00 ± 1.73	4.680	0.011
Communication anxiety	9.64 ± 2.57	9.87 ± 2.68	14.00 ± 1.00	4.095	0.019
Literature reading anxiety	13.72 ± 3.05	14.00 ± 3.70	18.00 ± 1,00	2.449	0.090
Essay writing anxiety	10.63 ± 2.87	10.83 ± 2.63	12.67 ± 2.08	0.833	0.437
Overall anxiety	60.45 ± 12.14	63.53 ± 14.25	83.00 ± 5.00	4.957	0.008

**TABLE 9:** Parameter estimation with low anxiety as a reference category.

Grouping^a^		B	*SE*	Wald	*df*	*p*	Exp(B)	95% confidence interval for exp(B)	
								Lower limit	Upper limit
High anxiety	Intercept	17.536	1550.085	0.000	1	0.991			
	[Geographical background = urban]	−1.738	0.701	6.149	1	0.013	0.176	0.045	0.695
	[Geographical background = rural]	0*^b^*	.	.	0	.	.	.	.
	[Grade = excellent]	−17.148	1550.085	0.000	1	0.991	3.572E − 08	0.000	.–
	[Grade = medium]	−16.489	1550.085	0.000	1	0.992	6.899E − 08	0.000	.–
	[Grade = fail]	0*^b^*	.	.	0	.	.	.	.
Medium anxiety	Intercept	1.553	0.477	10.597	1	0.001			
	[Geographical background = urban]	−1.385	0.510	7.355	1	0.007	0.250	0.092	0.681
	[Geographical background = rural]	0*^b^*	.	.	0	.	.	.	.
	[Grade = excellent]	0.781	0.510	2.341	1	0.126	2.183	0.803	5.937
	[Grade = medium]	0.619	0.000	.	1	.	1.857	1.857	1.857
	[Grade = fail]	0*^b^*	.	.	0	.	.	.	.

^a^The reference category is low anxiety; ^b^Reference category.

According to the data in Table 8, medical students with excellent English proficiency scored lower in all dimensions of English learning anxiety than poor English learners or average students. Among them, there were significant differences in the dimensions of vocabulary learning anxiety (*F* = 6.100, *p* = 0.003 < 0.05), and listening and speaking anxiety were significantly different (*F* = 4.680, *p* = 0.011 < 0.05). The scores were significantly different in communication anxiety (*F* = 4.095, *p* = 0.019 < 0.05), and there were significant differences in overall anxiety (*F* = 4.957, *p* = 0.008 < 0.05). There was no significant difference in either literature reading anxiety or academic writing anxiety.

### Multiple logistic regression analysis

The above studies showed that there were significant differences in anxiety scores at different levels of the geographical background and language achievement variables, so we used logistic regression to further determine the relationship between these two factors and anxiety, that is, whether the geographical background and test scores affect the degree of medical English learning anxiety. The model likelihood ratio test results were significant (χ^2^ = 24.16, *p* = 0.000 < 0.001), indicating that the regression analysis is effective.

According to the data in Table 9, when comparing high anxiety with low anxiety, geographical background had a significant effect on high anxiety or low anxiety (*p* = 0.013 < 0.05). The specific influence relationship is Logit (P high anxiety/P low anxiety) = −1.738 (geographical background = urban), B = −1.738 Exp(−1.738) = 0.176. From the regression equation, it can be seen that medical students with two backgrounds have a possibility of high anxiety, which is 0.176 times that of students from rural areas. Above all, medical students with rural backgrounds are more likely to have high anxiety in medical English learning.

Comparing moderate anxiety with low anxiety, geographical background had a significant effect on moderate or low medical English learning anxiety (*p* = 0.007 < 0.01). The specific influence relationship is Logit (Pan anxiety/P low anxiety) = −1.385 (geographical background = urban) B = −1.385 Exp (−1.385) = 0.250. From the regression equation, it can be seen that medical students from urban areas have a possibility of moderate English learning anxiety, which is 0.250 times that of medical students in rural areas. This indicates that rural medical students are more likely to have moderate anxiety in English learning.

As seen from Table 10, comparing moderate anxiety with high anxiety, English scores had a significant effect on medical students’ moderate anxiety or high anxiety (*p* = 0.000 < 0.001). From the regression equation, it can be seen that medical students with excellent medical English scores had a possibility of moderate anxiety rather than high anxiety. Comparing low anxiety with high anxiety, medical English scores have a significant impact on medical students’ low anxiety or high anxiety. Medical students with excellent medical English scores are more likely to have low anxiety in medical English learning. Overall, the better the medical English scores are, the lower the anxiety generated in medical English learning.

**TABLE 10:** Parameter estimation with high anxiety as a reference category.

Grouping^a^		B	*SE*	Wald	*df*	*p*	Exp(B)	95% confidence interval for exp(B)	
								Lower limit	Upper limit
Medium anxiety	Intercept	−16.983	0.349	2364.282	1	0.000			
	[Geographical background = urban]	0.354	0.567	0.388	1	0.533	1.424	0.468	4.331
	[Geographical background = rural]	0*^b^*	.	.	0	.	.	.	.
	[Grade = excellent]	18.928	0.506	1398.358	1	0.000	1.66E + 08	6.16E + 07	4.48E + 08
	[Grade = medium]	18.108	0.000	.	1	.	7.32E + 07	7.32E + 07	7.32E + 07
	[Grade = fail]	0*^b^*	.	.	0	.	.	.	.
Low anxiety	Intercept	−18.536	0.546	1154.420	1	0.000			
	[Geographical background = urban]	1.738	0.701	6.149	1	0.013	5.686	1.440	22.462
	[Geographical background = rural]	0*^b^*	.	.	0	.	.	.	.
	[Grade = excellent]	18.148	0.661	753.694	1	0.000	7.61E + 07	2.08E + 07	2.78E + 08
	[Grade = medium]	17.489	0.000	.	1	.	3.94E + 07	3.94E + 07	3.94E + 08
	[Grade = fail]	0*^b^*	.	.	0	.	.	.	.

^a^The reference category is high anxiety; ^b^Reference category.

## Discussion

Medical English learning matters greatly in China’s college English education today. English counts more for Chinese medical students than any other majors. Developing a specific scale aimed at medical majors is necessary. We designed 18 items in MELAS and examined overall reliability and validity. Cronbach’s alpha analysis revealed that the internal consistency reliability of this scale was very high in this study (alpha = 0.886, *n* = 5). The first research question examined the overall situation of the participants’ medical English anxiety. The participants reported that regarding the overall situation of their medical English anxiety, (85.2%) of the students surveyed had a moderate score and above, the proportion of which was higher than that of the participants tested by FLCAS (83.2%) (Deng and Zhou, 2018). This suggests that more medical students suffered moderate above anxiety by MELAS. Ke and Wang (2017) ascribed the reason as teachers with both professional medical knowledge and high English proficiency are insufficient in China and most teachers are not able to explain professional vocabulary and phrases due to the lack of English or professional knowledge which affects students’ interest and enthusiasm in medical English, causing and contributing to their language learning anxiety.

Among all five dimensions, students with listening and speaking anxiety accounted for the highest proportion (89.3%), followed by literature reading anxiety (86.6%), English academic writing anxiety (85.9%), communication anxiety (81.9), and vocabulary learning anxiety (81.2%). Consistent with previous research, Chinese college students are weak in English listening and speaking. You (2020) pointed out that due to the lack of good English phonetic ability and cross-cultural ability, many non-English majors have low levels of English listening and speaking. The participants’ reading and writing anxiety ranked second and third, respectively, which indicates that literature reading and paper writing in medical English are difficult for most medical students. One possible explanation is that medical English has long and complex professional terms and long complex sentence structures that are difficult to remember. A large number of medical English words come from Greek and Latin. In addition, the meaning of a word in general English usually has other specific explanations related to medicine as a profession, for instance, *positive*, *capsule*, *shock*, *piles*, *tender*, *remove*, and *regimen*. To achieve compact writing and objective and rigorous expressions, medical English has a certain pattern in syntax. It is characterized by more nominals, more active voice and long complex sentences. Due to the extensive use of nominals, clauses or non-finite structures derived from verbs in sentences, it increases the difficulty of understanding and makes Chinese learners fear reading and writing (Shi and Gan, 2012).

The second research question focused on the effects of biographical variables on FLA. First, there were no significant differences between male and female participants’ MELA which confirms past research findings (Dewaele et al., 2008; Garcia de Blakeley et al., 2017). For a long time, there has been no consensus on differences in language anxiety in males and females. For example, some studies found that females reported higher anxiety levels than males (Ezzi, 2012; Park and French, 2013); while other findings hold that male participants’ anxiety is higher than that of females (Wang, 2003). A possible explanation for the contradictory results may be there are other socio-biographical factors which also play a role in participants’ anxiety in addition to gender. It is hard to say that who are more anxious in a foreign language learning context, females or males, as well as there might be other factors, such as age of English language acquisition, one’s personality, self-confidence, and attitude.

Second, geographical background had a significant effect on the participants’ MELA. In this study, the anxiety of rural medical students in each dimension was higher than that of urban medical students. This echoes the finding of Yan and Horwitz (2008) who found that Chinese students from rural areas suffer more FLA than their counterparts from economically developed regions. In addition, Piechurska-Kuciel (2012) found that rural students suffer from significantly higher levels of anxiety over the length of their secondary school education when compared to their urban and suburban peers in Polish secondary grammar school. Xu (2016) provided two reasons as why rural students suffered higher anxiety than urban students. Firstly, the unbalanced distribution of educational resources is a fact in China’s urban and rural education; that is, a large number of high-quality teaching resources are available in abundance in cities; while teaching resources in rural areas are relatively scarce. Secondly, from the perspective of students’ growth experience, rural students normally grow up in more traditional villages, and it is generally believed that rural students are more introverted and sensitive in personality. This kind of difference in the learning environment and growth environment affects students’ anxiety level in learning English in China.

The third research question examined the links between participants’ medical English anxiety and language achievement. The effect of language anxiety on students’ achievement and performance in English language learning has been a concern in language anxiety studies. Numerous studies suggested a negative correlation between English language learning and language anxiety (Woodrow, 2006; Amiri and Ghonsooly, 2015; Cakici, 2016; Ali and Fei, 2017; Ozer and Akayolu, 2021). The negative relationship between medical English anxiety in vocabulary, listening and speaking, and communication and language achievement was expected as confirmed in the above-mentioned previous studies. Some researchers provided reasons behind such a negative relation. If a student feels anxious in the classroom, the possibility of having a frustrating and bad experience with the foreign language increases (Gregersen and Horwitz, 2002). Ewald (2007) posited that language learners, who experience language anxiety, are more worried about failing a course. Fear of interacting with native speakers, giving oral presentations, and performing in front of classmates are also sources of anxiety. In addition, language anxiety is a strong indicator of negative attitudes toward language learning (Woodrow, 2006).

## Conclusion

The present study investigated the MELA of Chinese medical university students by using self-developed MELAS and explored the relationships between biographical variables and their MELA. In general, moderate MELA existed among the participants. The proportion of participants (85.2%) tested by MELAS to be more anxious in medical English learning was higher than that of students (83.2%) tested by FLCAS. Geographical background has an effect on MELA overall; while gender has no effect on MELA. Multiple logistic regression analysis was further conducted to determine the relationship between geographical background, language achievement and anxiety, and it was found that geographical background and test scores affect the degree of medical English learning anxiety.

The contribution of this study is that in the past research, relevant studies generally only considered the relationship between academic achievement and anxiety in regression analysis, but not categorical variables as demographic variables. In addition, previous studies generally used a College English Test (Band 4) or College English Test (Band 6) score to represent language achievement, and the scores of one examination cannot represent the students’ real level in China. This study used logistic regression, and the scores of the dependent variables (excellent, average, and fail) better reflect the real learning capacity of the students.

Limitations of this study lie in the small sample. The sample should be bigger to generalize the study. Future studies can focus on students from other majors by using an adapted scale according to their major features and research diverse factors, including students’ personality, self-confidence, and self-perception of L2 competence.

## Data availability statement

The raw data supporting the conclusions of this article will be made available by the authors, without undue reservation.

## Author contributions

JD designed the research and drafted, wrote, and revised the manuscript. KZ analyzed all the data by using SPSS and AMOS. GA-S commented on the development of the manuscript, revised, edited, and proofread the manuscript. All authors contributed to the article and approved the submitted version.
