# Supplementary material for: Medical English anxiety patterns among medical students in Sichuan, China
Source: Front Psychol. 2022 Aug 10;13:895117. doi: 10.3389/fpsyg.2022.895117 (PMC9399909; doi:10.3389/fpsyg.2022.895117)
Supplement: Supplementary file 1 [file Data_Sheet_1.docx]

**Appendix A Chinese version of the adapted scale**

医学专业英语学习焦虑调查问卷

您好!为了解医学生在医学英语学习方面的焦虑情况，我们设计了以下的问卷。本研究采用不记名方式，调查结果只用于汇总后的统计分析，不会给您带来任何负面的影响。回答没有对错，请如实作答。感谢您的支持与配合。

请在符合您个人情况的选项前打√。

第一部分 个人基本信息

1. 您的性别： 男 女

2. 您来自： 城镇 农村

3. 您的年龄段： 大一 大二 大三 大四

4. 您医学专业英语的成绩是：80-90 60-80 60以下

第二部分 医学英语焦虑情况

（①非常不同意，②不同意，③不一定， ④同意， ⑤非常同意）

1. 一想到医学专业英语词汇就头痛

非常不同意 非常同意

① ② ③ ④ ⑤

2. 医学英语词汇很难记，使我很苦恼

非常不同意 非常同意

① ② ③ ④ ⑤

3. 医学英语学习没有以往学习英语的乐趣

非常不同意 非常同意

① ② ③ ④ ⑤

4.听不懂老师说什么我很慌张

非常不同意 非常同意

① ② ③ ④ ⑤

5. 对于医学英语口语摸不着头脑我很慌张

非常不同意 非常同意

① ② ③ ④ ⑤

6. 医学英语专业性太强说起来很吃力

非常不同意 非常同意

① ② ③ ④ ⑤

7. 我对用英语进行面对面交流很没有信心

非常不同意 非常同意

① ② ③ ④ ⑤

8. 医学专业英语听力太难，我很担心英语听力考试

非常不同意 非常同意

① ② ③ ④ ⑤

9. 每逢要上专业英语课就紧张

非常不同意 非常同意

① ② ③ ④ ⑤

10. 即使准备了课⇄发言也紧张

非常不同意 非常同意

① ② ③ ④ ⑤

11. 我觉得其他同学阅读英文文献的能力比我强

非常不同意 非常同意

① ② ③ ④ ⑤

12. 外文文献专业词汇太多令我抓狂

非常不同意 非常同意

① ② ③ ④ ⑤

13. 不懂他们为何害怕阅读英文文献

非常不同意 非常同意

① ② ③ ④ ⑤

14. 如果可能我尽量不用英文文献

非常不同意 非常同意

① ② ③ ④ ⑤

15. 我很期待与外国人交流

非常不同意 非常同意

① ② ③ ④ ⑤

16. 和外国人交流我很兴奋

非常不同意 非常同意

① ② ③ ④ ⑤

17. 听力考试时我紧张得会的内容都没听出来

非常不同意 非常同意

① ② ③ ④ ⑤

18. 一看到外国人一想到要和他说话就紧张得不知说什么

非常不同意 非常同意

① ② ③ ④ ⑤

19. 想都不敢想写医学英语学术论文

非常不同意 非常同意

① ② ③ ④ ⑤

20. 在写英文论文时我寝食难安

非常不同意 非常同意

① ② ③ ④ ⑤

21. 在别人面前说英语会面红耳赤

非常不同意 非常同意

① ② ③ ④ ⑤

22. 英文文献中很多生词会使我很恼火以至于影响阅读

非常不同意 非常同意

① ② ③ ④ ⑤

23. 阅读中连续遇到生词会使我很恼火以至于不想读下去

非常不同意 非常同意

① ② ③ ④ ⑤

24. 我觉得阅读中有不懂的很正常

非常不同意 非常同意

① ② ③ ④ ⑤

25. 我觉得最难的就是英语学术论文写作

非常不同意 非常同意

① ② ③ ④ ⑤

**Appendix B Translated version of the adapted scale**

Medical English Language Anxiety Questionnaire

To understand the anxiety of medical students in medical English learning, we designed the following questionnaire. This study adopts an anonymous method, and it will not bring you any negative impact because the survey results are only used for statistical analysis. There is no right or wrong answer. Please answer truthfully. Thank you for your support and cooperation.

Please tick √ before the options that meet your personal situation.

Part I Basic personal information

1. Your gender: A. male B. female

2. You are from: A. urban areas B. rural areas

3. You are a: A. freshman B. sophomore C. junior D. senior

4. Your score of medical English test is: A. 80-90 B. 60-80 C. under 60

Part II Medical English anxiety

(① very disagree, ② disagree, ③ not sure, ④ agree, ⑤ very agree)

1. It is a headache when mentioning medical English vocabulary

very disagree very agree

1. ② ③ ④ ⑤

2. Medical English vocabulary is difficult to remember, which makes me very distressed

very disagree very agree

① ② ③ ④ ⑤

3. Medical English learning is not as fun as learning English in the past

very disagree very agree

① ② ③ ④ ⑤

4. I am very nervous when I do not understand what the teacher is talking

very disagree very agree

① ② ③ ④ ⑤

5. I'm flurried when I feel at a loss about oral medical English

very disagree very agree

① ② ③ ④ ⑤

6. Medical English is too professional and difficult to speak

very disagree very agree

① ② ③ ④ ⑤

7. I have no confidence in face-to-face communication in English

very disagree very agree

① ② ③ ④ ⑤

8. Medical English listening is too difficult. I'm worried about the English listening test

very disagree very agree

① ② ③ ④ ⑤

9. I get nervous every time I have to take a medical English class

very disagree very agree

① ② ③ ④ ⑤

10. I will be nervous even if I have prepared for my speech in class

very disagree very agree

① ② ③ ④ ⑤

11. I think other students are better than me in reading medical English literature

very disagree very agree

① ② ③ ④ ⑤

12. Too many professional terms in foreign medical literatures drive me crazy

very disagree very agree

① ② ③ ④ ⑤

13. I do not understand why they are afraid of reading English literature

very disagree very agree

① ② ③ ④ ⑤

14. If possible, I try not to use English literature

very disagree very agree

① ② ③ ④ ⑤

15. I look forward to communicating with foreigners

very disagree very agree

① ② ③ ④ ⑤

16. I'm very excited to communicate with foreigners

very disagree very agree

① ② ③ ④ ⑤

17. In the English listening test, I even don’t understand what I am listening which I should have known because I am so nervous.

very disagree very agree

① ② ③ ④ ⑤

18. When I see a foreigner, I am too nervous to say anything at the thought of talking to him

very disagree very agree

① ② ③ ④ ⑤

19. I cannot even think of writing medical English academic papers

very disagree very agree

① ② ③ ④ ⑤

20. I had trouble sleeping and eating when I was writing my medical English academic paper

very disagree very agree

① ② ③ ④ ⑤

21. I will be flushed when speaking English in front of others

very disagree very agree

① ② ③ ④ ⑤

22. Many new words in medical English literature will annoy me and affect my reading

very disagree very agree

① ② ③ ④ ⑤

23. Nonstop new words in medical English literature reading will make me so angry that I do not want to keep reading it

very disagree very agree

① ② ③ ④ ⑤

24. I think it is normal to read something I do not understand in literature reading

very disagree very agree

① ② ③ ④ ⑤

25. I think the most difficult part is medical English academic paper writing

very disagree very agree

1. ② ③ ④ ⑤
